# Both *Neisseria gonorrhoeae* and *Neisseria sicca* Induce Cytokine Secretion by Infected Human Cells, but Only *Neisseria gonorrhoeae* Upregulates the Expression of Long Non-Coding RNAs

**DOI:** 10.3390/pathogens11040394

**Published:** 2022-03-24

**Authors:** Jagoda Płaczkiewicz, Monika Adamczyk-Popławska, Ewa Kozłowska, Agnieszka Kwiatek

**Affiliations:** 1 Department of Molecular Virology, Institute of Microbiology, Faculty of Biology, University of Warsaw, 02-096 Warsaw, Poland; jplaczkiewicz@ichf.edu.pl (J.P.); poplawa@biol.uw.edu.pl (M.A.-P.); 2 International Centre for Translational Eye Research, Institute of Physical Chemistry, Polish Academy of Sciences, 01-224 Warsaw, Poland; 3 Department of Immunology, Institute of Functional Biology and Ecology, Faculty of Biology, University of Warsaw, 02-096 Warsaw, Poland; ekozlowska@biol.uw.edu.pl

**Keywords:** *Neisseria gonorrhoeae*, *Neisseria sicca*, gene expression, long non-coding RNA, cytokines, chemokines

## Abstract

Bacteria of the *Neisseria* genus are Gram-negative diplococci including both pathogenic and commensal species. We focused on pathogenic *Neisseria gonorrhoeae* and commensal *Neisseria sicca*. We have demonstrated that not only *N. gonorrhoeae*, but also *N. sicca* induce the secretion of pro-inflammatory cytokines IL-6, TNF-α, and chemokines CXCL8 and CCL20 by infected epithelial cells. However, *N. sicca* triggers a lesser effect than does *N. gonorrhoeae*. Furthermore, *N. gonorrhoeae* and *N. sicca* invoke distinct effects on the expression of genes (JUNB, FOSB, NFKB1, NFKBIA) encoding protein components of AP-1 and NF-κB transcription factors. We have also shown that the infection of epithelial cells by *N. gonorrhoeae* leads to significant overexpression of the long non-coding RNAs (lncRNAs), including MALAT1, ERICD, and RP11-510N19.5. This effect was not identified for *N. sicca.* In conclusion, data on the expression of lncRNAs and cytokine secretion in response to *Neisseria* spp. exposure indicate new directions for research on *Neisseria*-host interactions and can provide further insights into virulence of not only pathogenic, but also commensal *Neisseria* spp.

## 1. Introduction

Bacteria of the *Neisseria* genus are Gram-negative diplococci, classified as β-proteobacteria. The most clinically significant members of this genus are the pathogenic species *Neisseria gonorrhoeae* and *Neisseria meningitidis*. *N*. *gonorrhoeae* (gonococcus) is a causative agent of gonorrhea, one of the oldest documented sexually transmitted diseases (STDs). Enduringly, gonococcal infections constitute a major global health issue, with more than 88 million cases reported annually worldwide [1]. In 2017, the World Health Organization listed *N. gonorrhoeae* as a “priority pathogen”, as gonococcal strains have already acquired resistance to many antimicrobials, including so-called last line option antibiotics like third-generation cephalosporins and azithromycin [2]. Additionally, inflammation elicited by *N. gonorrhoeae* in infected tissue with subsequent disruption of mucosal integrity increases susceptibility to HIV type I infections [3]. This bacterium may also exert a powerful amplifying factor in increasing the risk of precancer state and cancer development in infected tissue [4].

In addition to pathogens, the *Neisseria* genus includes many less well-studied species, usually referred to as “commensal”, inter alia, *Neisseria lactamica*, *Neisseria elongata*, *Neisseria sicca,* or *Neisseria cinerea,* constituting the natural human microbiome [5]. These commensals, living in continuous interaction with the human mucosa, have evolved various mechanisms to ensure colonization and maintenance of host-bacteria balance. Colonizing human mucous membranes, commensals can also influence the impact of pathogenic bacteria on host cells. For example, it was shown that commensal *N. elongata* kills *N. gonorrhoeae* in vitro and accelerates the clearance of gonococci from mice hosts. Further, the DNA of the commensal species of *Neisseria* kills *N. meningitidis* [6]. On the other hand, commensal *Neisseria* spp. can be opportunistic pathogens and have the potential to cause disease, particularly in immunodeficient hosts [7,8,9,10,11]. Regardless of these data, the interactions of commensal *Neisseria* spp. with host cells have been, until now, poorly investigated.

Recently, non-coding RNAs (ncRNAs), including small ncRNAs and long ncRNAs (lncRNAs), have been pointed out as important factors in the response reaction of host cells to pathogens [12]. Initially, small ncRNAs were intensively studied in this field. Currently, the studies are turning towards lncRNAs [13,14,15]. To date, most research on the involvement of lncRNAs in defending against infectious agents mainly concerned viruses [16]. Data on the lncRNAs in mammalian cells exposed to bacteria are limited, including cells exposed to commensal or pathogenic *Neisseria* spp.

The aim of our research was the estimation and comparison of the cytokines secretions by human epithelial cells subjected to pathogenic *N. gonorrhoeae* and commensal *N. sicca.* We also determined the induction ability of lncRNA expression by the pathogenic and commensal *Neisseria* sp. during the infection of human epithelial cells.

## 2. Results and Discussion

### 2.1. N. gonorrhoeae and N. sicca Induce an Immune Response in Human Epithelial Cells

**Figure 1:**
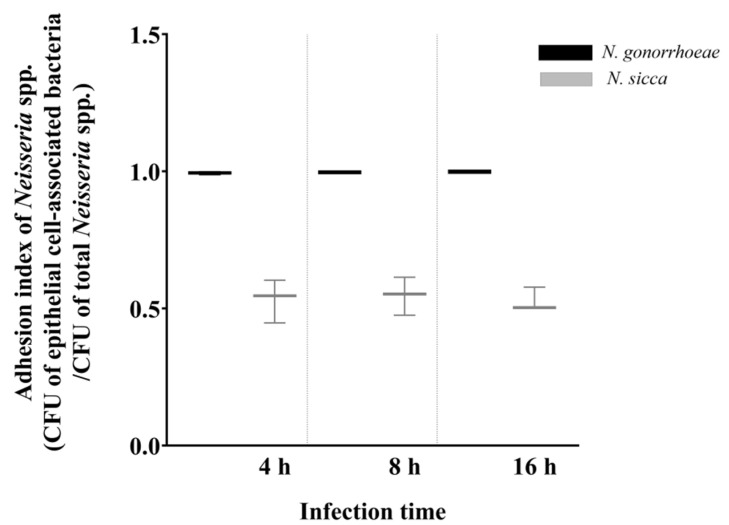
The adhesion of *Neisseria* spp. to human epithelial cells. The adhesion was determined as an adhesion index value, which was measured as the colony forming unit (CFU) of epithelial-associated *Neisseria* spp. divided by the total CFU of *Neisseria* spp. The data are presented as the mean values from at least three experiments performed in duplicate (±SD).

The immune response towards infecting bacteria requires initial adhesion of microorganisms to host cells. The evaluation of adhesion levels of *N. gonorrhoeae*, and *N. sicca*, was performed after 4, 8, and 16 h of infection with the same multiplicity of infection (MOI). Obtained results indicated that both bacteria can adhere to our model epithelial cells in all tested periods of infection, while *N*. *sicca* is characterized by a lower adhesion level in comparison to *N. gonorrhoeae* (Figure 1).

The results concerning *N. gonorrhoeae* adhesion are in line with other data, including those indicating the interactions of gonococci with male and female urogenital epithelia [17,18,19]. Nonetheless, infections of other organs and tissue by gonococci, such as the conjunctiva, pharynx, and rectal mucosa are also reported [20]. To what extent commensal *Neisseria* spp. exhibit tropism for their hosts is largely unknown and so far, in vitro models for the study of the impact of commensal *Neisseria* spp. on host cells are not described.

**Figure 2:**
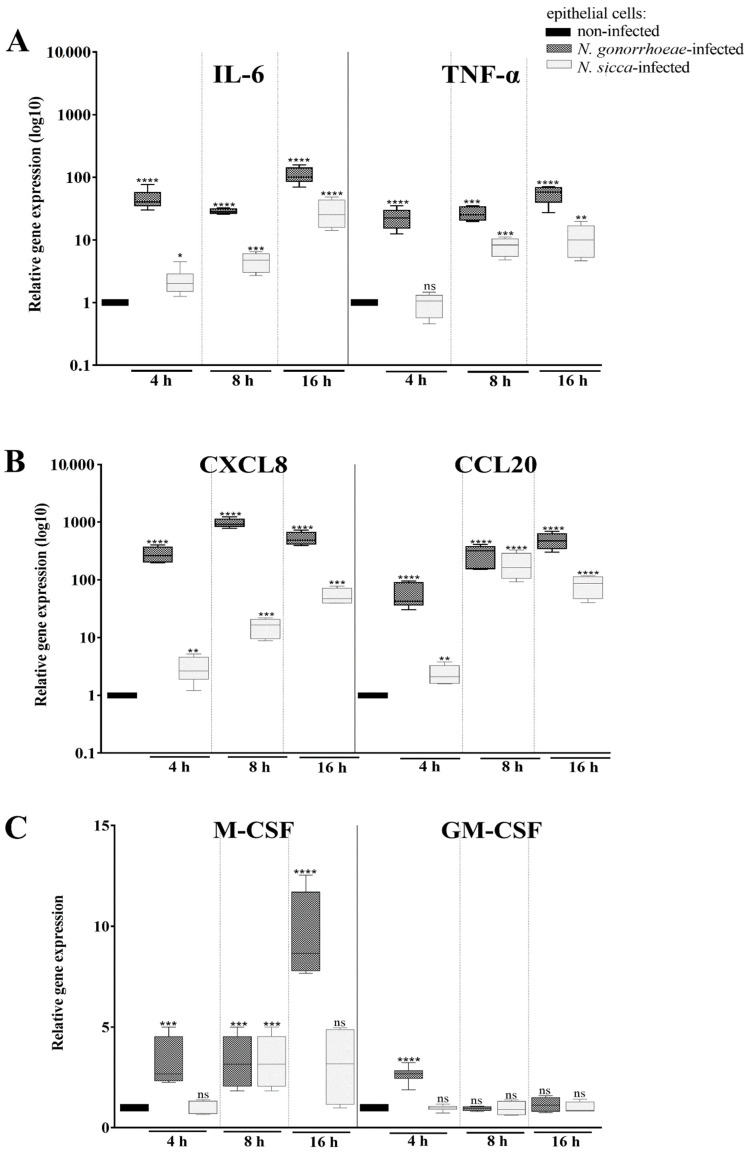
The expression of cytokine genes is affected by *N. gonorrhoeae* and *N. sicca* infection. Panel (**A**) the expression of pro-inflammatory cytokines; (**B**) of chemotactic cytokines; (**C**) of colony stimulating factors (CSFs). The gene expression after 4, 8, and 16 h of bacterial infection was evaluated by RT-qPCR, followed by a relative quantitation data analysis using the ΔΔCt comparative quantification method. The gene expression in epithelial cells infected by *Neisseria* spp. was compared to that observed in non-infected epithelial cells, for which a value of 1 was assumed. Significant differences in gene expression in epithelial cells subjected to *Neisseria* spp. compared to that observed in non-infected epithelial cells are marked by asterisks: * *p* < 0.05, ** *p* < 0.005, *** *p* < 0.0005 and ****; *p* < 0.0001; ns—not statistically significant. The data are presented as the mean values from at least three experiments performed in duplicate (±SD).

As mentioned above, the contact of microorganisms with host cells triggers the immune response. To study the immune response of human epithelial cells subjected to pathogenic *N*. *gonorrhoeae* or commensal *N*. *sicca*, first, the expression of genes encoding key pro-inflammatory cytokines (IL-6, TNF-α), chemotactic cytokines (CCL20 and CXCL8), and colony stimulating factors (CSFs) was measured using RT-qPCR. As presented in Figure 2, epithelial cells exposed to *N. gonorrhoeae* or *N. sicca* differentially expressed the genes of the mentioned cytokines as compared to non-infected human cells. However, the transcriptional response of epithelial cells to *N. sicca* was impaired in comparison to the response elicited by *N. gonorrhoeae*. After 4 h of infection, the expression of cytokines encoding genes IL-6, CCL20, and CXCL8 was increased in human cells infected by either *N. gonorrhoeae* or by *N. sicca* compared to expression in the non-infected cells (41.8-, 45.7-, and 176.9-fold, respectively, for cells infected by *N. gonorrhoeae* and 2.6-, 1.88-, and 2.2-fold for cells infected by *N. sicca*). However, the TNF-α, M-CSF, and GM-CSF genes were overexpressed only in epithelial cells subjected to *N. gonorrhoeae*, but not in those subjected to *N. sicca*. The expression of these genes increased 20.9-, 2.55-, and 2.39-fold, respectively, in *N. gonorrhoeae*-infected cells compared to non-infected cells.

After 8 and 16 h of infection, *Neisseria* spp. still caused overexpression of the IL-6, TNF-α, CCL20, and CXCL8 genes. In turn, the expression of the M-CSF gene was increased after 8 and 16 h of *N. gonorrhoeae* infection. After 8 h of contact, *N. sicca* also increased the expression of the M-CSF gene (2.1-fold overexpression) in epithelial cells. After 16 h of exposure to commensal *Neisseria* sp., the differences in expression of M-CSF gene were not statistically significant, while infection by *N. gonorrhoeae* caused an 9.37-fold overexpression of the M-CSF gene. The expression of the GM-CSF gene was not affected by either *N. gonorrhoeae* or *N. sicca* after 8 and 16 h of infection (Figure 2).

**Figure 3:**
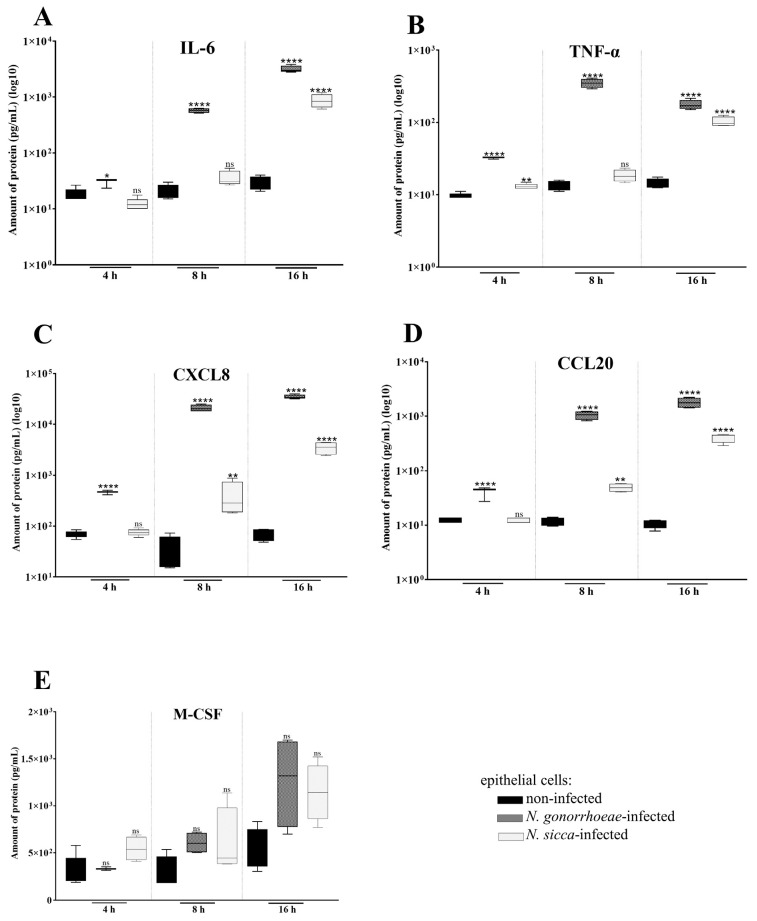
*N. gonorrhoeae* and *N. sicca* induce distinct cytokine secretion by human epithelial cells. The amount of pro-inflammatory cytokines (**A**,**B**), chemotactic cytokines (**C**,**D**), and macrophage colony stimulating factor (**E**) was evaluated by flow cytometry after 4, 8, and 16 h of infection. The concentration of cytokines is given as pg/mL. Significant differences in the amount of cytokines secreted by epithelial cells subjected to *N. gonorrhoeae* or *N. sicca* compared to that secreted by non-infected epithelial cells are indicated by asterisks: * (*p* < 0.05), ** ( *p* < 0.005), and **** (*p* < 0.0001); ns—not statistically significant. The data are presented as the mean values from at least three experiments performed in duplicate (±SD).

To evaluate whether the noticed overexpression of the above genes also results in an increase of secreted proteins, the amount of the cytokines was measured by flow cytometry. After 4 h of exposure of human epithelial cells to studied *Neisseria* spp., the significant differences in the amount of secreted cytokines by *N. gonorrhoeae*-infected or *N*. *sicca*-infected cells compared to non-infected cells were also observed (Figure 3).

Detailed comparative analysis of the amount of IL-6, CCL20, and CXCL8 proteins secreted by *N*. *gonorrhoeae*-infected and non-infected human cells showed significant statistical differences (*p* < 0.05). *N. gonorrhoeae*-infected cells secreted 2.42-, 3.28-, and 6.69-fold more of these cytokines, respectively, compared to non-infected human cells. In turn, there were no significant differences for secretion of IL-6, CCL20, and CXCL8 between non-infected and *N. sicca*-infected human cells (*p* > 0.05). This suggests that commensal *N. sicca* did not activate the secretion of these cytokines after 4 h of infection, unlike *N*. *gonorrhoeae*. For the cytokine M-CSF, no statistically significant differences were observed between non-infected and *N. gonorrhoeae*- or *N. sicca*-infected human cells at this time point.

To estimate whether differences in protein secretion observed in human epithelial cells subjected to *Neisseria* spp. for 4 h persist after 8 and 16 h of infection, the levels of these cytokines after these periods time were also examined (Figure 3).

After 8 h of infection, the level of TNF-α and IL-6 secreted by *N. gonorrhoeae*-infected human cells was 18.39- and 16.13-fold higher, respectively, compared to non-infected cells. In contrast, these cytokines were not statistically significantly higher in those secreted by epithelial cells subjected to commensal *N. sicca* in comparison to non-infected human cells. In turn, in comparison to non-infected cells, secretory concentrations of CXCL8 and CCL20 were found to be significantly increased in human cells after infection with *N. gonorrhoeae*, and modestly, but still statistically relevantly, elevated in cells subjected to *N. sicca*. As after 4 h of infection, the concentration of M-CSF secreted by human cells subjected to *N. gonorrhoeae* or to *N. sicca* was not statistically significant for the checkpoint of 8 h.

After 16 h of infection, both bacteria induced a statistically significantly higher synthesis of the cytokines by human cells, compared to synthesis by non-infected human cells. *N*. *sicca* stimulated an increase in the secretion of TNF-α, IL-6, CXCL8, and CCL20 by 7.17-, 29.58-, 50.62-, and 37.24-fold in comparison to non-infected human cells, respectively. Those observations indicate that contact with *N. sicca* had an impact on the physiology of epithelial cells; whereas, the epithelial cells infected by *N. gonorrhoeae* secreted 12.36-, 107.01-, 501.61-, and 163.07-times more TNF-α, IL-6, CXCL8, and CCL20 than non-infected cells. The amount of M-CSF secreted by both bacteria-treated human cells was not significantly different compared to those by non-infected cells, the same as after 4 and 8 h of infection.

Our data on cytokine secretion in response to *N. gonorrhoeae* infection in vitro are consistent with previously published results indicating the release of pro-inflammatory cytokines and chemokines IL-6, CXCL8, and TNF-α during symptomatic infection in humans [21,22]. Secretion of TNF-α and CXCL8 by epithelial cells in response to invasion by other bacteria, e.g., *Salmonella* spp., *Shigella dysenteriae*, *Yersinia enterocolitica*, *Listeria monocytogenes*, and enteroinvasive *Escherichia coli*, has also been documented [23]. Thus, such activation of cytokine production is not only specific to pathogenic gonococci, but also seems to be a more general response to pathogen infection.

The available data on immune response stimulation after contact of human cells with commensal *Neisseria* spp., both in vitro and in vivo, are limited and mainly related to *N. lactamica*. For example, porin PorB expressed by *N. lactamica* was demonstrated to act as a low inducer of CXCL8, TNF-α, and IL-6 secretion in human airway epithelial cells [24,25]. Additionally, for commensal *Neisseria musculi* it was demonstrated that IL-6 receptor-deficient mice were efficiently colonized by these bacteria, indicating host IL-6 production as a critical factor in determining host colonization susceptibility [26]. There is no data on host immune response after contact with *N. sicca*. We demonstrate that commensal *N. sicca* can induce cytokine secretion by human epithelial cells in vitro, but the secretion level of these cytokines is lower than that triggered by pathogenic *N. gonorrhoeae*. This may suggest that the activation of IL-6, CXCL8 and TNF-α secretion is not specific only to pathogenic *Neisseria* spp., but appears to be a general response of human cells to *Neisseria* spp. exposure, including commensal types.

The expression and secretion of CXCL8 and TNF-α have also been elicited by non-pathogenic *E. coli* and *Lactobacillus sakei* in CaCO-2 epithelial cells in co-culture with leukocytes [27]. However, the level of activation of cytokine secretion is different for human cells affected by commensal and pathogenic species. According to Haller D. et al. (2000), this activation can depend on the difference in a network of interactions between host epithelium and leukocytes [27].

CXCL8 and CCL20 are key chemotactic cytokines. CXCL8 primarily induces the chemotaxis of neutrophils, and CCL20 is strongly chemotactic for lymphocytes, weakly attracting neutrophils and causing leukocyte migration toward the site of infection [28]. The highly increased secretion of the chemoattractants CXCL8 and CCL20 by *N*. *gonorrhoeae*-infected epithelial cells, in each time interval examined, suggests that gonococcal infection induces a strong chemotactic response towards immune cells, and the recruitment of immune cells is likely to result in the development of inflammation. In contrast, the increase in CCL20 and CXCL8 secretion by *N. sicca*-infected human cells was noticed only after 16 h, unlike to *N. gonorrhoeae*-infected cells, in which the effect was observed after only 4 h of infection. This result does not exclude the possibility of leukocyte chemotaxis at the site of commensal bacteria attachment to host cells, but this process would be attenuated and delayed compared to that induced by pathogenic bacteria.

Chemotaxis and the activation of immune cells stimulated by CXCL8 and CCL20 are crucial during inflammation and are critical for the clearance of pathogens. Activated neutrophils migrate to the infection site, where they form the first cellular line of defense. On the other hand, the increased secretion of CXCL8 and CCL20 may contribute directly to the inflammatory signs and symptoms characteristic of disease caused by *N. gonorrhoeae*. Thus, CXCL8 may be clinically used as a biomarker of inflammatory processes. In turn, recent results of Sanyal A. et al. (2019) suggest that an increase in CXCL10 and CXCL8 secretion in epithelia may be responsible for *N. gonorrhoeae*-induced enhanced HIV-1 transmission across cervical mucosa [29].

### 2.2. Expression of AP-1 and NF-κB Transcription Factors Is Affected by the Presence of N. gonorrhoeae and N. sicca

**Figure 4:**
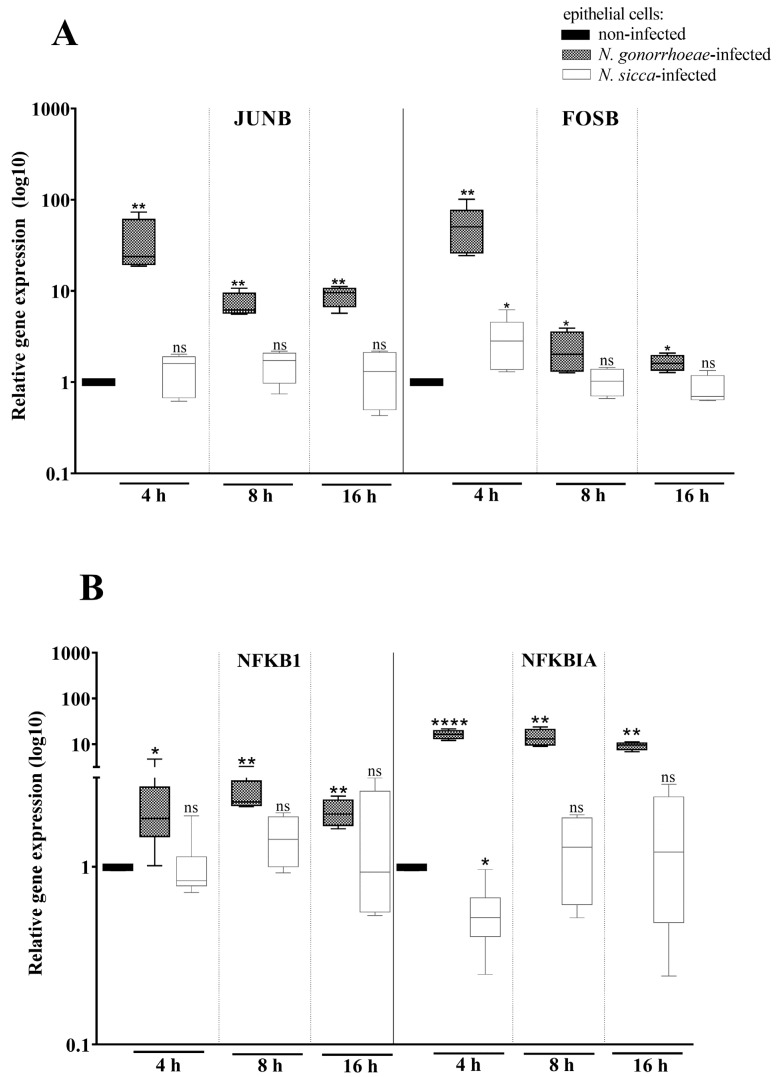
The exposure of human cells to *N. gonorrhoeae* or *N. sicca* causes the differential gene expression of components of AP-1 (**A**) and NF-κB (**B**) transcription factors. Gene expression was estimated by RT-qPCR, and then a relative quantitation data analysis was performed using the ΔΔCt comparative quantification method. The gene expression in epithelial cells infected by *Neisseria* spp. was compared to that observed in non-infected epithelial cells, for which a value of 1 was assumed. Significant differences in gene expression in epithelial cells subjected to *Neisseria* spp. compared to that observed in non-infected epithelial cells are marked by asterisks: * *p* < 0.05, ** *p* < 0.005, and **** *p* < 0.0001; ns—not statistically significant. The data are presented as the mean values from at least three experiments performed in duplicate (±SD).

The expression of genes encoding immune system proteins is tightly regulated by transcription factors, including AP-1 and NF-κB. AP-1 is a heterodimer composed of JunJUN and FosFOS proteins, and the main structural element of the NF-κB complex is a p50 protein encoded by the NFKB1 gene [30,31]. Here, we have studied the expression of genes encoding these proteins and the NFKBIA gene encoding an inhibitor of NF-κB (IκBα) in human cells after 4, 8, and 16 h of contact with *N*. *gonorrhoeae* or *N. sicca* (Figure 4).

The expression of JUNB and FOSB genes was increased 24.51- and 36.06-fold in *N*. *gonorrhoeae*-infected human cells compared to non-infected cells after 4 h of infection. What is more, as presented in Figure 4, not only *N. gonorrhoeae* caused overexpression of FOSB, but *N. sicca* was also able to stimulate FOSB overexpression compared to non-infected human cells (a 2.38-fold increase of expression). However, the level of JUNB expression was not statistically significantly affected by *N. sicca* compared to non-infected human cells. Thus, we cannot rule out that proper AP-1 complexes are not formed or even absent in *N. sicca*-infected cells after 4 h of infection. In turn, after 8 and 16 h of exposure of epithelial cells to *N. gonorrhoeae* or *N. sicca*, only gonococci caused the overexpression of genes encoding components of the AP-1 transcription factor. It suggests that *N. gonorrhoeae* can activate the AP-1 for a longer period of time as compared to *N. sicca*.

As presented in Figure 4, the infection of human epithelial cells with *N. gonorrhoeae* results in a 1.96-fold increase of NFKB1 expression, encoding p50 protein, while treatment of human cells by *N. sicca* did not induce differential expression of this gene compared to non-infected cells. In turn, the NFKBIA gene encoding IκBα inhibitor of NF-κB was 17-fold overexpressed and 2-fold down-expressed, respectively, in *N*. *gonorrhoeae*-infected and *N. sicca*-infected cells compared to non-infected human cells. However, the effect was observed only after 4 h of infection. After 8 and 16 h, the expression of NFKB1 and NFKBIA genes was affected only by *N. gonorrhoeae*.

The results presented on *N. gonorrhoeae* are in line with the published data demonstrating the role of NF-κB and AP-1 signaling in transcription regulation of TNF-α and IL-6 in gonococcal-infected human cells [17]. This supports the observation on the disturbance of the NF-κB pathway by *N. gonorrhoeae* infection.

Likewise for the immune response, the available data on the expression of transcription factors after contact of human cells with commensal *Neisseria* spp. concern only to *N. lactamica,* which can decrease NF-κB activity, priming immunological ignorance. Until now, there was no data related to the impact of *N. sicca* on transcriptional regulation and cellular signaling. Our data indicated the regulation of expression of IκBα inhibitor of NF-κB as a response to contact of epithelial cells with *N. sicca*. This phenomenon, similar to that observed in *N. lactamica,* may play a role in host-microbe homeostasis and the immune privilege of commensal species.

### 2.3. Long Non-Coding RNAs Are Differentially Expressed Mostly in Human Cells Treated by N. gonorrhoeae Compared to Those Treated by N. sicca

Non-coding RNAs have been identified as pivotal regulators of gene expression at every stage of gene expression and cell functioning, including signaling pathways related to immune response [32]. Today, no data concerning lncRNAs during *N. gonorrhoeae* infection are available. Therefore, we estimated whether infection of human cells by pathogenic and commensal bacteria from *Neisseria* spp. influences the expression of ncRNAs.

**Figure 5:**
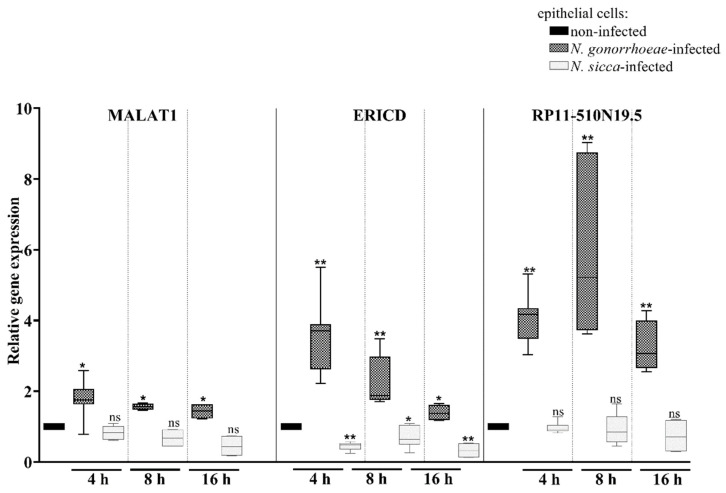
The infection of epithelial cells by *N. gonorrhoeae* and *N. sicca* results in the overexpression of long non-coding RNAs. Gene expression, after 4, 8, and 16 h of bacterial infection, was estimated using RT-qPCR, followed by a relative quantitation data analysis using the ΔΔCt comparative quantification method. The gene expression in epithelial cells infected by *Neisseria* spp. was compared to that observed in non-infected epithelial cells, for which a value of 1 was assumed. Significant differences in gene expression in epithelial cells subjected to *Neisseria* spp. compared to that observed in non-infected epithelial cells are marked by asterisks: * *p* < 0.05 and ** *p* < 0.005; ns—not statistically significant. The data are presented as the mean values from at least three experiments performed in duplicate (±SD).

As presented in Figure 5, *N. gonorrhoeae* induced the overexpression of MALAT1, ERICD, and RP11-510N19.5 genes encoding lncRNAs, by 2.7-, 3.31-, and 4.12-fold, respectively, when compared to non-infected human cells after 4 h of infection. No differential expression of MALAT1 and RP11-510N19.5 genes was observed in *N. sicca*-infected cells compared to non-infected human cells, while the expression of ERICD was decreased 2-fold. After 8 and 16 h of infection, the observed differential expression of the lncRNAs persisted. It should be noted that among the studied lncRNAs, the expression of RP11-510N19.5 was the most affected in gonococcal infected epithelial cells in each considered infection period.

MALAT1, ERICD, and RP11-510N19.5 belong to lncRNAs; the first two represent a subtype of lncRNA called lincRNAs, and the latter is a sense intronic.

Despite the emerging interest in the role of lncRNAs in cellular signaling, most data on the lncRNAs concerning their role in the crosstalk between bacteria and host cells have been obtained from cells induced only with purified lipooligosaccharide (LPS). To our knowledge, the presented results are the first data on the expression of lncRNAs in epithelial cells exposed to *Neisseria* spp., and one of few existing studies concerning lncRNAs expression in human cells infected by live bacteria; e.g., the differential expression of MALAT1 has been previously shown in LPS-stimulated monocytes/macrophages, where it negatively regulates the expression of inflammatory cytokines (TNF-α and IL-6) by influence on the activity of NF-κB [33,34,35]. In turn, our data demonstrated the increased expression of MALAT1 in human cells exposed to live bacteria. Further, this effect was observed in epithelial cells treated by pathogenic, but not by a commensal, bacterium.

The second lncRNA—RP11-510N19.5—the expression of which was increased in pathogen-, but not in commensal-exposed epithelial cells, was not studied before in any context. Our in silico analysis revealed that the RP11-510N19.5 gene is located within the gene encoding an ELF3 transcription factor that binds and transactivates external transcribed spacer (ETS) sequences in the promoter of the gene encoding CCL20 chemokine. In turn, the third lncRNA considered in our studies, ERICD, has not been studied before in the context of the host–pathogen interactions. Previously, it was demonstrated that it participates in the cellular response to DNA damage and the regulation of migration and proliferation of osteosarcoma cells [36]. Our in silico analysis revealed that the ERICD gene is associated with the gene of argonaute-2 (AGO2) protein, which is required for RNA-mediated gene silencing by the RNA-induced silencing complex (RISC). AGO2 binds to the AU element of the 3′-UTR of the TNF-α mRNA and up-regulates TNF-α translation. These data might imply the commitment of MALAT1, ERICD, and RP11-510N19.5 in cellular response, in particular in immune response, mainly against pathogens, but not commensals. It is consistent with data on the role of other lncRNAs as, e.g., NEAT1, lincRNA-Cox2, lncRNA-CD244, MEG3, lncRNA Sros1, lncRNA AS-IL1α, and lnc-GNAT1, whose differential expression was connected to infections caused by *Helicobacter pylori*, *Mycobacterium tuberculosis*, *Mycobacterium smegmatis*, *Mycobacterium bovis*, *Listeria monocytogenes* and influencing the secretion of inflammatory factors or cell fate (apoptosis or autophagy) [37,38,39,40,41,42,43,44,45]. The identification of putative lncRNAs candidates among deregulated genes in bovine macrophages after *Mycobacterium avium* subspecies *paratuberculosis* infections [46] also confirms our observations on the commitment of lncRNAs in a mammalian cells response to living bacteria.

Nonetheless, it is intriguing that the ERICD expression was increased in epithelial cells exposed to the pathogen, but decreased in the commensal-treated cells. To integrate these data with the knowledge about the potential effects of ERICD on TNF-α secretion, it should be noted that the secretion of TNF as a mature protein is tightly regulated at the transcriptional, posttranscriptional, translational, and posttranslational levels. The control of the activation of TNF-α gene expression at the level of transcription is only the first step in TNF synthesis. Different factors, including TNF enhanceosome, which is cell type- and stimulus-specific, involving distinct sets of transcription factors and coactivators, control TNF expression [47]. Thus, ERICD may be one, but not the only, regulator of TNF-α gene transcription. Our observations suggest the involvement of the ERICD in cellular response against both pathogens and commensals by different mechanisms. Another possible explanation might be related to the engagement of ERICD in the immune privilege of commensal bacteria within its host, although this requires further examination.

Thus, our data outline new research directions for the study of pathogenic and commensal *Neisseria* spp., including *N. sicca*.

## 3. Materials and Methods

### 3.1. Bacterial Culture

*N. gonorrhoeae* FA1090 (ATCC: 700825™) and *N. sicca* AMC 14-D-1 (DSMZ: 23539) were grown at 37 °C and 5% CO_2_, on GC agar base (Difco, Detroit, MI, USA) supplemented with 1% Kellogg’s supplement, and depending on the experiment, with or without 1% hemoglobin. For all experiments, gonococci exhibiting the same piliation and opaque phenotype were used, as determined by microscopy observation in accordance with the principles described by Dillard J.P. (2011) [48]. Prior to each experiment, inoculum of a predominantly Pili^+^ Opa^+^ frozen stock of *N. gonorrhoeae* FA1090 was spread and cultivated for 24 h on GC agar base without hemoglobin to evaluate gonococcal colony morphologies under a stereo dissecting microscope. Next, Pili^+^ and opaque phenotype colonies were picked, streaked on GC agar base supplemented with hemoglobin, and cultivated for the next 24 h.

### 3.2. Human Cell Culture and Infection

The human epithelial cell line HEC-1-B (ATCC: HTB113), was used for bacterial infection [46,49]. The HEC-1-B cells were cultured in DMEM (Biowest, Nuaille, France) supplemented with 10% fetal bovine serum (Biowest) (*v*/*v*), 2 mM L-glutamine, 1 mM sodium pyruvate, and incubated at 37 °C and 5% CO_2_. For bacterial infection, a suspension of *N. sicca* or *N. gonorrhoeae* (1 mL of 10^8^ cells/mL suspension, prepared in supplemented DMEM) was added to 10^6^ epithelial cells cultured on a 35 mm culture dish (multiplicity of infection: 100 bacterial cells to 1 epithelial cell (M.O.I. 1:100)), and cultures were continued at 37 °C and 5% CO_2_ for 4, 8, or 16 h.

### 3.3. Adhesion Assays of Neisseria spp. to Human Epithelial Cells

The adhesion assay was performed as described previously by Płaczkiewicz J. et al., 2019, and Płaczkiewicz J. et al., 2020 [49,50]. To this purpose, human epithelial cells were cultured to confluence, which was followed by infection with *Neisseria* spp. for 4, 8, and 16 h (MOI of 1:100). Subsequently, one set of the *Neisseria* spp.-infected epithelial cells was washed with PBS, lysed by 0.5% saponin, and the lysates were plated on GC agar base with 1% hemoglobin for enumeration of the colony forming unit (CFU) of cell-associated gonococci. At the same time, another set of the *Neisseria* spp.-infected epithelial cells was not rinsed with PBS, but only the culture medium from the above cells was removed; the epithelial cells were lysed by 0.5% saponin and then plated on GC agar base with 1% hemoglobin for the enumeration of CFU. The sum of CFU from the culture medium and from the cell lysates constitutes the total CFU. The level of *Neisseria* spp. adhesion was calculated by determining the adhesion index value, which was estimated as the CFU of the epithelial-associated *Neisseria* spp. divided by the total CFU of the *Neisseria* spp.

### 3.4. RNA Extraction

Total RNA from: (i) non-infected epithelial cells; (ii) *N. gonorrhoeae*-infected epithelial cells; (iii) *N. sicca*-infected epithelial cells was extracted using a Total RNA Mini Kit (A&A Biotechnology, Gdynia, Poland). The bacterial infection of the epithelial cells was conducted for 4, 8, and 16 h, as described above. The contaminating DNA was removed using the TURBO DNA-free™ Kit (Thermo Fisher Scientific, Waltham, MA, USA). A 2100 Bioanalyzer (Agilent Technologies, Santa Clara, CA, USA) was used to measure the RNA concentration and quality, and RNA with an RNA integrity number (RIN) of >8.0 was used in subsequent assays.

### 3.5. Real-Time RT-qPCR

Real-time PCR experiments were performed according to the Minimum Information for Publication of Quantitative Real-Time PCR Experiments guidelines [51]. The cDNAs were obtained by reverse transcription of 10 µg of total RNA using the RevertAid First Strand cDNA Synthesis Kit (Thermo Fisher Scientific). Real-time PCR using HOT FIREPol^®^ EvaGreen^®^ qPCR Mix Plus (ROX) (Solis BioDyne, Tartu, Estonia) was performed on a LightCycler^®^ 96 system (Roche, Basel, Switzerland) (95 °C for 900 s, 40 cycles of 95 °C for 15 s, 60 °C for 30 s, and 72 °C for 20 s). Oligonucleotide primer pairs specific for each human gene of interest were obtained from Bio-Rad (Hercules, CA, USA) or Thermo Fisher Scientific (Supplementary data, Table S1). Relative quantification of gene expression was performed using the comparative ΔΔCt (threshold cycle) method. The relative amount of target cDNA was normalized using the *Hypoxanthine phosphoribosyltransferase 1* (*HPRT*) gene and *Beta-2-microglobulin* (*B2M*) gene for human gene expression.

### 3.6. Cytokine Assay

The amount of cytokine was measured by flow cytometry. To this purpose, culture media from epithelial cells exposed to *N. gonorrhoeae* or *N. sicca* were collected after 4, 8, and 16 h of infection. Simultaneously, culture media from non-infected human cells were collected and used as a control. Then, cytokines were assayed using custom-designed LEGENDplex bead arrays (BioLegend, San Diego, CA, USA) following the manufacturer’s recommendations. Data acquired by flow cytometry (FACSVerse, BD Biosciences, Franklin Lakes, NJ, USA) were analyzed by LEGENDplex Data Analysis Software v8 (BioLegend). All supernatants, including replicates from three independent experiments, were assayed in duplicate.

### 3.7. Statistical Analysis

The differences in gene expression and the amount of cytokines secreted were calculated using two-way ANOVA followed by Bonferroni posttests. Data include results from at least three experiments performed in duplicate.

## 4. Conclusions

Comparison of the level of cytokines secreted by human cells subjected to commensal and pathogenic *Neisseria* sp. might point to immune response as another important factor contributing to bacterial pathogenicity. On the other hand, data on the immune response to *N. sicca* exposure permits the consideration of how this commensal triggers inflammation in exposed human cells, e.g., by eliciting the secretion of pro-inflammatory and chemotactic cytokines.

Our data also suggest the role of long non-coding RNAs as important players in the cross-talk between pathogenic *Neisseria* and host cells. Specifically for pathogenic, but not for commensal *Neisseria* sp., the overexpression of lncRNAs may be one of the determinants of pathogenicity by influencing the immune response and inflammation.

Thus, our investigations provide further insights into *Neisseria* pathogenesis and indicate directions for research on *Neisseria*-host interactions.

## Data Availability

Not applicable.

## Supplementary Materials

The following supporting information can be downloaded at: https://www.mdpi.com/article/10.3390/pathogens11040394/s1, Table S1: Primers used for RT-qPCR.
