# Supplementary material for: Both Neisseria gonorrhoeae and Neisseria sicca Induce Cytokine Secretion by Infected Human Cells, but Only Neisseria gonorrhoeae Upregulates the Expression of Long Non-Coding RNAs
Source: Pathogens. 2022 Mar 24;11(4):394. doi: 10.3390/pathogens11040394 (PMC9031631; doi:10.3390/pathogens11040394)
Supplement: Supplementary file 1 [file pathogens-11-00394-s001.zip › pathogens-1585030-supplementary.pdf]

**Supplementary Table S1.** Primers used for RT-qPCR.

| **Gene** | **Primer sequence (5' → 3') /catalog number/assay ID** |
|---|---|
| CCL20 | 10025636*<br>qHsaCID0011773** |
| IL-6 | 10025636*<br>qHsaCID0020314** |
| CXCL8 | 10025636*<br>qHsaCED0023767** |
| TNF-α | 10025636*<br>qHsaCED0037461** |
| M-CSF | 10025636*<br>qHsaCED0047665** |
| GM-CSF | 10025636*<br>qHsaCED0002766** |
| JunB | 10025636*<br>qHsaCED0018781** |
| FosB | 10025636*<br>qHsaCED0023033** |
| NFKBIA | 10025636*<br>qHsaCED0002729** |
| NFKB1 | for: AGGGCCACTGAGCACTATTT<br>rev: AATAGCAGAGGCTCCAGGTG |
| MALAT1 | for: AAGACCTCGACACCATCGTTAC<br>rev: CTAAGGTCAAGAGAAGTGTCAG |
| ERIC | for: GTCTGCATATGGGTGCAAGG<br>rev: TTCTCCTGCCCTTCTTCAGG |
| RP11-510N19.5 | 12004175*<br>qhsaLED0105915** |
| B2M | 10025636*<br>qHsaCID0015347** |
| HPRT | 4326321E***<br>Hs9999909m1**** |

* catalogue number (Bio-Rad Laboratories)

** assay ID (Bio-Rad Laboratories)

*** catalogue number (Thermo Fisher Scientific)

**** assay ID (Thermo Fisher Scientific)
